# Experiencia del desarrollo de herramientas para el monitoreo de coberturas de vacunación y quimioterapia preventiva

**DOI:** 10.26633/RPSP.2017.141

**Published:** 2017-12-20

**Authors:** Martha Idalí Saboyà-Díaz, Ana Morice, M. Carolina Danovaro-Holliday, Cuauhtémoc Ruiz Matus, Luis Gerardo Castellanos, Martha Patricia Velandia-Gonzáez

**Affiliations:** 1 Organización Panamericana de la Salud Organización Panamericana de la Salud Washington D.C. Organización Panamericana de la Salud, Washington D.C., Estados Unidos de América.

**Keywords:** Enfermedades desatendidas, monitoreo, cobertura de vacunación, Neglected diseases, in all monitoring, immunization coverage, Doenças negligenciadas, monitoramento, cobertura vacinal

## Abstract

**Objetivo.:**

*Describir la experiencia y las lecciones aprendidas en el desarrollo de herramientas para el monitoreo de las coberturas de vacunación y de quimioterapia preventiva en las Américas*.

**Métodos.:**

*Se compiló y revisó la documentación disponible en los programas regionales de inmunización integral de la familia y de las enfermedades infecciosas desatendidas de la Organización Panamericana de la Salud/Organización Mundial de la Salud producida durante el proceso de desarrollo de las herramientas entre 2012 y 2017 incluidos: búsqueda sistemática de literatura, reportes técnicos, informes de reuniones internas, reporte de la prueba piloto e informes y resultados de las evaluaciones de los talleres de entrenamiento en doce países de las Américas. La documentación se organizó, se extrajo la información más relevante sobre el desarrollo de las herramientas y se consensuaron las principales lecciones aprendidas en el proceso*.

**Resultados.:**

*El proceso facilitó la organización y sistematización de las herramientas aplicadas durante las últimas dos décadas en los programas de vacunación en las Américas para el análisis y monitoreo de las coberturas y su rápida adaptación para el monitoreo de las coberturas de la quimioterapia preventiva. Se integró un conjunto de herramientas cuya aplicación se define con un algoritmo de decisiones y pueden ser utilizadas por los países*.

**Conclusiones.:**

*Las lecciones aprendidas pueden aplicarse para desarrollos similares y para promover el trabajo interprogramático en salud pública. El trabajo integrado aceleró el desarrollo de herramientas útiles para los países*.

## Abstract (en)

**Objective.:**

Describe the experience and lessons learned in the development of tools to monitor vaccination coverage and preventive chemotherapy coverage in the Americas.

**Methods.:**

The available documentation on Pan American Health Organization/World Health Organization regional programs for comprehensive family immunization and neglected infectious diseases––produced during the process of tool development from 2012 to 2017––was compiled and reviewed, including: systematic literature search, technical reports, internal meeting reports, pilot study report, and reports and results from evaluations of training workshops in 12 countries of the Americas. The documentation was organized, gleaning the most relevant information on tool development, and consensus was reached on the principal lessons learned in the process.

**Results.:**

The process facilitated the organization and dissemination of tools used to analyze and monitor vaccination coverage during the last two decades in vaccination programs in the Americas, and the rapid adaptation of these tools for monitoring preventive chemotherapy coverage. A toolkit was put together, accompanied by a decision tree to aid in its application, which countries can use.

## RESUMO

**Objetivo.:**

Descrever a experiência e as lições aprendidas no desenvolvimento de ferramentas para o monitoramento da cobertura vacinal e quimioprofilaxia nas Américas.

**Métodos.:**

Foi coletada e revisada a documentação disponível nos programas regionais de imunização integral da família e doenças infecciosas negligenciadas da Organização Pan-Americana da Saúde/Organização Mundial da Saúde, produzida no processo de desenvolvimento de ferramentas entre 2012 e 2017. Os arquivos englobavam resultados de busca sistemática da literatura, relatórios técnicos, atas de reuniões internas, informe de teste-piloto e relatórios e resultados das avaliações dos seminários de capacitação em 12 países das Américas. A documentação foi organizada e foram extraídos os dados mais relevantes sobre o desenvolvimento das ferramentas. As principais lições aprendidas no processo foram decididas por consenso.

**Resultados.:**

O processo facilitou a organização e a sistematização das ferramentas empregadas nas últimas duas décadas nos programas de vacinação nas Américas para avaliação e monitoramento da cobertura vacinal e sua rápida adaptação para o monitoramento da cobertura da quimioprofilaxia. Foi integrado um conjunto de ferramentas cuja aplicação é definida com um algoritmo de decisões e que pode ser utilizado pelos países.

**Conclusões.:**

As lições aprendidas podem ser aplicadas em processos semelhantes de desenvolvimento e para promover o trabalho interprogramático em saúde pública. O trabalho integrado acelerou o desenvolvimento de ferramentas úteis para os países.

La cobertura de vacunación es un indicador de desempeño de los programas de vacunación, en el que se divide el número de personas vacunadas por la población meta de un área geográfica específica, y se expresa en porcentaje. Este indicador se utiliza como trazador de la protección inmunológica adquirida para las enfermedades prevenibles por vacunación. La fuente del dato del numerador es el registro de personas vacunadas y la del denominador es la población estimada, en general, con base en las proyecciones de los censos oficiales. Esta forma de cálculo se conoce como cobertura administrativa o reportada de vacunación (1, 2).

Durante las últimas dos décadas se han desarrollado herramientas para monitorear y evaluar las coberturas de vacunación, entre las que se hallan los monitoreos rápidos de coberturas de vacunación que aplican metodologías sencillas y rápidas para identificar grupos de población no vacunados con el fin de vacunarlos de forma inmediata (3, 4), las encuestas de base poblacional para estimar las coberturas de vacunación mediante métodos probabilísticos para generar un dato robusto de las coberturas (5, 6) y el análisis de la calidad del dato que evalúa la exactitud de los datos reportados de personas vacunadas en un período de tiempo determinado, la oportunidad e integralidad del reporte y la capacidad del sistema de información para recolectar, transmitir, documentar, reportar, monitorear y evaluar datos de vacunación de calidad (7, 8).

La cobertura de la quimioterapia preventiva (QP), que es el uso de medicamentos antiparasitarios o antibióticos solos o combinados para controlar o eliminar las enfermedades infecciosas desatendidas (EID) en poblaciones endémicas (9), es el número de personas que recibieron tratamiento del total de personas que lo requieren en un área geográfica endémica para la enfermedad específica, y se expresa en porcentaje (10). Este es un indicador que el programa de las EID utiliza para monitorear el desempeño con el fin de tener información sobre el progreso en la interrupción de la transmisión de una enfermedad. La QP se usa para reducir la prevalencia de enfermedades como, por ejemplo, las geohelmintiasis al tratar a los grupos de población en riesgo de infectarse como los preescolares o escolares mediante la administración masiva de medicamentos (11, 12). En el 2010, la Organización Mundial de la Salud (OMS) recomendó a los países hacer el monitoreo rápido de coberturas de la QP mediante el uso de las metodologías desarrolladas por los programas de vacunación (10).

La Organización Panamericana de la Salud (OPS), a través de los programas regionales de inmunización integral de la familia y de las enfermedades infecciosas desatendidas en las Américas, realizó en el 2012 una revisión de la literatura en busca de evidencia sobre el uso de métodos para el monitoreo de coberturas de vacunación y desparasitación y encontró que la difusión de experiencias publicadas al respecto era limitada (13). Esto llevó a que los dos programas regionales decidieran desarrollar herramientas que facilitaran el monitoreo de coberturas de las dos intervenciones por separado o en forma conjunta. Se presentan a continuación la experiencia y las lecciones aprendidas en el proceso.

## MATERIALES Y MÉTODOS

Para describir la experiencia del desarrollo de las herramientas para el monitoreo de coberturas de vacunación y QP, se revisaron los siguientes documentos elaborados entre el año 2012 y agosto del 2017 en los programas regionales de inmunización integral de la familia y de las EID de la OPS/OMS: 1) resultados de búsqueda sistemática de metodologías para el monitoreo de coberturas para vacunación y desparasitación (13), 2) resultados de la revisión de metodologías que los países de las Américas han empleado para el monitoreo de coberturas de vacunación del programa de rutina y de las campañas, 3) los resúmenes y ayudas de memoria de las reuniones entre delegados de los dos programas de la OPS/OMS en las que se establecieron los acuerdos sobre los contenidos y estructura de las herramientas para el monitoreo de coberturas, 4) los resultados de la prueba piloto de las herramientas de monitoreo de coberturas realizada en Nicaragua (14, 15), 5) los borradores y diversas versiones de los documentos con las herramientas desarrolladas entre 2012 y 2017 y 6) los informes y evaluaciones de los participantes de los doce talleres realizados en países de las Américas para la capacitación en la aplicación de las herramientas.

La información del proceso de desarrollo de las herramientas se organizó con criterio cronológico y se extrajo la información más relevante. Se identificaron, discutieron y consensuaron las leccines aprendidas entre los delegados de los dos programas regionales.

## RESULTADOS

### Desarrollo del primer documento con las herramientas para el monitoreo de coberturas

En el 2012, los programas regionales de inmunización integral de la familia y de las EID de la OPS/OMS iniciaron el desarrollo de un protocolo para el monitoreo de coberturas de vacunación y de desparasitación para las geohelmintiasis con base en la vasta experiencia del programa de inmunizaciones en las Américas en este tema. En esta fase inicial, se escogió específicamente la desparasitación para el control de las geohelmintiasis porque varios países de la Región vacunaban y desparasitaban de forma simultánea en preescolares y escolares durante la Semana de Vacunación de las Américas (16–19).

El proceso se inició con la búsqueda sistemática de la literatura publicada desde 1990 hasta el año 2011 con relación al monitoreo tanto las coberturas de vacunación como de desparasitación en menores de 5 años (prescolares) y de 5 a 14 años (escolares). Se construyó una base de datos con 91 documentos revisados de un total de 729 identificados en diversas fuentes de búsqueda. Se encontró que solo 2 de los 91 documentos revisados reportaron un monitoreo conjunto y la metodología documentada con mayor frecuencia fue la encuesta: 77,8% (n = 14 de un total de 18 publicaciones) eran encuestas de coberturas de desparasitación y 78,1% eran de coberturas de vacunación (n = 57 de un total de 73 publicaciones) (13).

Por lo anterior, se definió la necesidad de elaborar un protocolo para el monitoreo conjunto de coberturas basado en las experiencias y metodologías utilizadas en el programa de vacunación en las Américas. Se recopilaron y sistematizaron las diferentes herramientas utilizadas para el monitoreo de coberturas del programa regular y en las campañas de vacunación y se analizaron sus ventajas y desventajas, así como las recomendaciones de expertos sobre su uso y alcance. Varios estudios y la experiencia del programa regional de inmunización resaltaban la necesidad de complementar los registros administrativos con monitoreos rápidos de coberturas, debido a que las coberturas administrativas o reportadas tienen limitaciones en sus numeradores y denominadores, que resultan de problemas en el flujo de información, errores en la compilación de datos en los diferentes niveles del sistema de reporte y reportes incompletos o tardíos, entre otros (20–22).

A fines del 2012, en una reunión de los delegados de los dos programas regionales, se decidió elaborar un protocolo que integrara un conjunto de herramientas complementarias entre sí para facilitar a los equipos de salud de cada uno de los ámbitos de gestión en salud de los países, el análisis y monitoreo de las coberturas de vacunación y QP a partir de criterios de decisión y procedimientos estandarizados. Estas herramientas debían facilitar a los países la revisión y análisis crítico de las coberturas administrativas o reportadas, la puesta en marcha de monitoreos rápidos de coberturas en escuelas y comunidades y la definición de criterios de decisión para planificar y ejecutar encuestas de coberturas, para lo cual debía tener las siguientes características:

1) Partir del uso sistemático de los datos de las coberturas administrativas o reportadas.
2) Organizar las herramientas de forma secuencial, con base en el uso de datos basados en coberturas administrativas, yendo de lo más sencillo a lo más complejo y por pasos para facilitar su uso y aplicación.
3) Incorporar criterios de decisión para seleccionar la herramienta a utilizar y orientar la toma de decisiones con base en los resultados obtenidos.
4) Incluir formatos para recopilar, presentar y divulgar los resultados.

### Prueba piloto

Al concluir el diseño de la primera versión del protocolo, se programó una prueba piloto para identificar necesidades de ajustes metodológicos y operativos e incorporar los cambios requeridos para que fuera de fácil comprensión, factible de aplicar en la práctica de los programas y útil para apoyar la toma de decisiones. La prueba piloto se llevó a cabo en Nicaragua en octubre del 2013 (14, 15). Este país fue seleccionado porque había desarrollado campañas nacionales de vacunación y desparasitación de niños menores de 15 años en las Semanas Nacionales de Salud Infantil durante más de 20 años y manifestó su interés en usar las herramientas propuestas por la OPS/OMS para el monitoreo de las coberturas.

Mientras se planificaba la prueba piloto, la OMS publicó el protocolo para la evaluación de la calidad del dato para las enfermedades infecciosas desatendidas (DQA por sus siglas en inglés de *data quality assessment*) que se acompañaba de una hoja de cálculo en Microsoft Excel®, una guía para el facilitador y ejercicios para el entrenamiento (23). Dado que la OMS contaba también con una herramienta para la autoevaluación de la calidad del dato de vacunación (DQS por sus siglas en inglés de *data quality self-assessment*) (7, 8), se decidió incluir esta herramienta en la prueba piloto. La incorporación del DQA y el DQS se consideró muy pertinente dada la relevancia de mejorar la calidad de los datos administrativos de dosis administradas y el análisis de los denominadores para el cálculo de las coberturas.

Para validar la propuesta metodológica se usaron los datos reales de Nicaragua y participaron delegados de los programas nacionales y locales de vacunación y desparasitación, delegados de los programas regionales de inmunización integral de la familia y de las EID de la OPS/OMS y un delegado del departamento de control de las enfermedades tropicales desatendidas de la OMS. Se analizaron las coberturas administrativas o reportadas de vacunación y de desparasitación del año inmediatamente anterior, se realizaron monitoreos rápidos de coberturas de vacunación y desparasitación casa a casa y en escuelas y se analizó la calidad de los datos de las dos intervenciones.

Los participantes de la prueba piloto resaltaron que las herramientas eran útiles porque ordenaban de manera sistemática varias metodologías e instrumentos que ya existían de forma individual en los programas de vacunación y además las adaptaba para su uso en la QP; la aproximación paso a paso fue práctica y fácil de seguir a nivel local; y la construcción basada en las experiencias de los países de la Región se resaltó como un valor agregado.

Con base en los resultados de la prueba piloto, se identificó la necesidad de: 1) desarrollar hojas de cálculo en Microsoft Excel® para facilitar la generación automática de cuadros y figuras de los resultados del análisis de las coberturas administrativas o reportadas, 2) incluir recomendaciones sobre el tipo de análisis de los datos de las coberturas administrativas o reportadas que se pueden hacer en cada uno de los niveles político-administrativos de un país (nacional, subnacional o local) y 3) desarrollar un cuaderno de capacitación para el facilitador y uno para el participante (15).

### Ajuste de las herramientas de monitoreo de coberturas después de la prueba piloto

**FIGURA 1.**
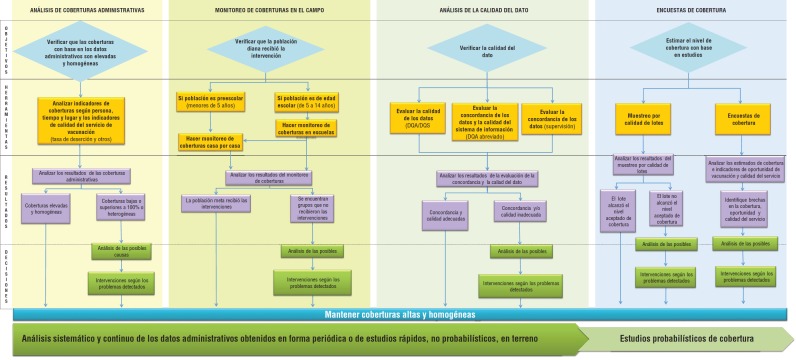
Descripción general de las herramientas para el monitoreo de las coberturas de intervenciones integradas en salud pública y algoritmo para su selección.

DQA, evaluación de la calidad del dato (por sus siglas en inglés); DQS, autoevaluación de la calidad del dato (por sus siglas en inglés).

Después de la prueba piloto, se produjo una versión ajustada del documento con las herramientas que se dividieron en dos categorías: 1) metodologías para el análisis sistemático y continuo de los datos administrativos obtenidos periódicamente o de estudios rápidos en terreno, no probabilísticos, que incluye las herramientas para el análisis de las coberturas administrativas o reportadas, el monitoreo de las coberturas en campo (comunidad y escuela) y el análisis de la calidad de los datos y 2) las herramientas para los estudios probabilísticos de cobertura que se enfocan en las encuestas de cobertura. En la figura 1 se presenta de forma gráfica la organización de las herramientas por objetivos y el algoritmo general para seleccionarlas.

Con el fin de facilitar el uso y aplicación de las herramientas a nivel local, el documento se organizó en seis módulos que se acompañan de un cuaderno para el facilitador, un cuaderno para el estudiante, presentaciones en Microsoft PowerPoint® y hojas de cálculo en Microsoft Excel®. Los diversos instrumentos se pueden adaptar a la realidad de cada país y nivel subnacional o local. Los contenidos de los seis módulos son los siguientes:

- Fundamentos conceptuales y metodológicos. Presenta los antecedentes que sustentan la pertinencia de sistematizar las herramientas para el monitoreo de las coberturas de vacunación y de la QP y usa como ejemplo de aplicación la desparasitación en los programas de control de las geohelmintiasis, a quién están dirigidos los módulos, sus usos y aplicaciones y la organización de sus contenidos. Este módulo tiene dos unidades: estrategias de intervención (vacunación y desparasitación para el control de las geohelmintiasis como ejemplo de las enfermedades en las que se usa la QP) y metodologías para el monitoreo de coberturas.
- Análisis de coberturas administrativas. Se describen los pasos recomendados para analizar las coberturas administrativas o reportadas de vacunación y desparasitación. Tiene dos unidades: coberturas administrativas de vacunación y coberturas administrativas de desparasitación para las geohelmintiasis. Se desarrollan los pasos para analizar tanto los datos absolutos como el porcentaje de cobertura, según tiempo, lugar y persona, la calidad de los servicios de vacunación y desparasitación (oportunidad, simultaneidad, deserción o abandono e integralidad), así como la calidad de los denominadores y numeradores.
- Monitoreo de coberturas en campo. Se describen las metodologías para hacer monitoreos rápidos de cobertura y tiene dos unidades: monitoreo rápido de coberturas casa por casa y monitoreo de coberturas en escuela.
- Análisis de la calidad de los datos. Este módulo tiene una sola unidad en la que se desarrolla la metodología de autoevaluación de la calidad de los datos (DQS) y se describen opciones metodológicas abreviadas de análisis de la calidad de los datos del programa de vacunación y desparasitación (DQA), que se aplican durante la supervisión de las campañas y los programas regulares.
- Encuestas de cobertura. Tiene dos unidades que describen cada uno de los pasos necesarios para planificar y realizar una encuesta de coberturas, mediante los dos métodos más comunes: el muestreo por conglomerados y muestreo por calidad de lotes.
- análisis de datos de encuestas y registros nominales electrónicos. Tiene una sola unidad que explica los pasos del análisis de los datos de las encuestas y registros nominales de vacunación electrónicos para obtención de indicadores, entre ellos, los elementos relativos a un plan y una estrategia de análisis, la verificación de la calidad de los datos, la aplicación de instrumentos de análisis descriptivo y modelación de los datos y la interpretación correcta de los resultados.

### Progreso en la aplicación: entrenamientos para el uso de las herramientas en las Américas

Antes de finalizar y publicar el documento con las herramientas, los dos programas regionales de la OPS/OMS decidieron desarrollar talleres de entrenamiento para delegados de países de las Américas de los programas de vacunación y de QP para las EID. Entre el 2015 y agosto del 2017, se llevaron a cabo doce talleres en los que se capacitaron 689 personas, con énfasis en el análisis de las coberturas administrativas, el monitoreo rápido de coberturas y la evaluación de la calidad de los datos para los programas de vacunación y de QP (cuadro 1).

Algunos resultados relevantes identificados por los participantes en estos entrenamientos fueron los siguientes:

- Las herramientas son flexibles porque los módulos se pueden usar por separado y los talleres de capacitación pueden hacerse para grupos de trabajadores de los servicios de salud en lugares en donde la vacunación y la QP se hacen de forma conjunta o independiente por cada programa.
- Los módulos y el material de soporte (cuadernos del facilitador y del estudiantes, las presentaciones y las hojas de cálculo) facilitan el uso de las herramientas para fortalecer las capacidades nacionales para el monitoreo de los programas de vacunación y de QP.
- Los entrenamientos introdujeron a los participantes en el uso de nuevas herramientas que deben complementar el análisis de las coberturas y que deben insertarse en la práctica de los servicios de salud de la Región, entre ellas el DQA y el DQS, mediante el uso de versiones completas y abreviadas.
- Los resultados de las prácticas de campo y del análisis de los datos propios en cada país se usaron para poner en marcha intervenciones para fortalecer el monitoreo de las coberturas en los programas de vacunación y de QP.
- La metodología de enseñanza y la aproximación práctica e interactiva de los talleres facilitaron el proceso de aprendizaje.
- La diversidad en el perfil de los participantes favoreció el trabajo en equipo y el intercambio de experiencias entre personas de diferentes disciplinas en el análisis e interpretación de los resultados.

**CUADRO 1.** Entrenamientos en el uso de las herramientas para el monitoreo de coberturas de intervenciones integradas en salud pública en países de las Américas, 2015-2017

País	Año y duración del taller	Tipo de taller	Participantes			Herramientas				Práctica de campo
			Procedencia^a^	Área de trabajo	Número	ACA	MR	DQS	EC	
México	2015, 3 días	Nacional	Nacional, subnacional y local	Vacunación	200	Sí	Sí	Sí	No	No
Honduras	2015, 3 días	Nacional	Nacional, subnacional y local	Vacunación y desparasitación	30	No	No	No	Sí	Sí
Honduras	2015, 3 días	Nacional	Nacional y subnacional	Vacunación, epidemiología, servicios de salud, estadística, estudiantes de epidemiología	70	Sí	No	No	No	No
El Salvador	2015, 4 días	Nacional	Nacional, subnacional y local	Vacunación, desparasitación, epidemiología y estadística	30	Sí	Sí	Sí	No	Sí
Honduras	2015, 5 días	Regional	Nacionales de Colombia, República Dominicana, Honduras, México, Nicaragua y Paraguay	Vacunación y desparasitación	30	Sí	Sí	Sí	No	Sí
Honduras	2016, 4 días	Nacional	Nacional y subnacional	Vacunación, epidemiología y estadística	62	Sí	Sí	Sí	No	Sí
Paraguay	2016, 4 días	Nacional	Nacional y subnacional	Desparasitación	20	Sí	Sí	Sí	No	Sí
Chile	2016, 3 días	Nacional	Nacional y subnacional	Vacunación	70	Sí	Sí	Sí	No	Sí
Guyana	2016, 5 días	Regional	Nacionales de Guyana y Surinam, locales de Guyana	Vacunación y filariasis linfática	30	Sí	Sí	Sí	No	Sí
Nicaragua	2017, 3 días	Nacional	Nacional, subnacional y local	Vacunación	67	Sí	Sí	No	No	No
Venezuela	2017, 2 días	Nacional	Nacional y subnacional	Vacunación Vacunación y filariasis	54	Sí	Sí	Sí	No	Sí
Haití	2017, 5 días	Nacional	Nacional y subnacional	linfática	26	Sí	Sí	Sí	No	Sí

a Nacional: ministerios de salud o instituciones nacionales responsables de los programas en salud pública en cada país; subnacional: estados, departamentos o provincias de acuerdo con la división político-administrativa de cada país; local: municipios en la mayoría de los casos.

ACA, análisis de coberturas administrativas; MR, monitoreos rápidos; DQS, autoevaluación de la calidad del dato; EC, encuestas de coberturas.

### Lecciones aprendidas

El proceso de desarrollo de las herramientas para el monitoreo de las coberturas de vacunación y QP ha sido sostenido y enriquecedor. En los diferentes talleres de entrenamiento y en las prácticas de campo desarrolladas con varios países se identificó la flexibilidad de las herramientas como una ventaja, toda vez que los instrumentos pueden adaptarse a las condiciones propias de cada país, a los grupos objetivo de las intervenciones y a las intervenciones en desarrollo. Por ejemplo, en Guyana y Haití se adaptaron las herramientas para el monitoreo de coberturas de la QP para eliminar la filariasis linfática y controlar las geohelmintiasis.

La publicación final del documento (en inglés y en español) está en proceso, aunque ya se han capacitado equipos de los programas de doce países de la Región de las Américas a través de los entrenamientos desarrollados. Una versión preliminar en español se puede encontrar en la página web del programa regional de inmunización integral de la familia de la OPS/OMS (24).

Son varias y diversas las lecciones aprendidas a lo largo de los años de desarrollo de las herramientas, las principales son:

- Este proceso refleja la importancia de compartir experiencias entre programas de salud pública, mediante el uso de metodologías, herramientas e instrumentos para monitorear coberturas de intervenciones que van dirigidas a grandes grupos de población a través de la plataforma de campañas, ya sea de vacunación o de QP. Ese trabajo conjunto requiere de personas que estén dispuestas a invertir tiempo y que tengan características personales como paciencia, persistencia y disposición para el trabajo en equipo.
- La búsqueda sistemática de metodologías de monitoreo de coberturas demostró que la literatura sobre este tema se enfoca sobre todo en la publicación de encuestas de cobertura. En ese sentido, el proceso de diseño y aplicación de las herramientas aprovechó las experiencias nacionales y regionales en el uso de las diversas metodologías y logró poner a disposición de los países un conjunto de herramientas prácticas para el monitoreo de las coberturas de dos programas de salud pública.
- El programa regional de las enfermedades infecciosas desatendidas ahorró tiempo y recursos en el desarrollo y validación de herramientas para el monitoreo de coberturas de la QP y se benefició de los desarrollos del programa de inmunización en las Américas, mientras que el programa regional de inmunización organizó y sistematizó sus herramientas para el monitoreo de coberturas.
- El trabajo interprogramático permitió compartir experiencias y reconocer la necesidad de facilitar el trabajo conjunto no solo a nivel regional sino a nivel nacional y local. Por ejemplo, el programa de inmunizaciones identificó que la implementación conjunta de acciones de vacunación con desparasitación para el control de las geohelmintiasis dirigidas a población concentrada en las escuelas promueve el incremento de coberturas de vacunación de población en edad escolar. Además, el programa de las enfermedades infecciosas desatendidas identificó la necesidad de impulsar en los países el desarrollo de registros y sistemas de información para monitorear las coberturas de la QP. Para ello, se aprovechó una vez más la experiencia del programa de inmunizaciones y está en proceso la publicación de un “Manual para formularios de registro de la quimioterapia preventiva para las enfermedades infecciosas desatendidas” (25).
- Los principales desafíos enfrentados en el desarrollo de las herramientas fueron: mantener el trabajo interprogramático a nivel regional como una constante, debido a que cada programa tiene sus propias agendas, tiempos y necesidades; llegar a consensos sobre las necesidades de cada programa, los conocimientos, las metodologías y las herramientas existentes, y comprender las particularidades de cada uno; y planificar y desarrollar entrenamientos porque se requiere tiempo y recursos desde los programas involucrados.
- El desarrollo de las herramientas no termina aún y se presentan retos futuros por ser un proceso en constante evolución. Uno de esos retos es sostener los entrenamientos para que se expanda el uso de las herramientas en la Región, en especial al nivel local; incorporar las herramientas en los componentes de monitoreo y evaluación de los programas de QP y de vacunación; mantener la herramienta actualizada, acorde al progreso de los programas, así como innovar en herramientas pedagógicas diseñadas para entrenamiento de adultos.

Como próximos pasos se requiere evaluar el uso, el costo y el impacto de la herramienta en los países de la Región y desarrollar metodologías de capacitación que no dependan exclusivamente de talleres presenciales que son demandantes en tiempo y costos, que pueden no ser sostenibles y que llegan a un número limitado de personas.

#### Agradecimientos

Los autores desean expresar su agradecimiento hacia Gina Tambini (exdirectora del departamento de Familia, Género y Curso de Vida de la OPS/OMS), Marcos Espinal (Director del Departamento de Enfermedades Transmisibles y Análisis de Salud de la OPS/OMS), Marcela Contreras (programa regional de inmunización integral de la familia-OPS/OMS), Laura Catalá y Ana Luciañez (programa regional de enfermedades infecciosas desatendidas), Nancy Vásconez (asesora en inmunizaciones de la oficina de la OPS/OMS en Nicaragua) y Aida Soto (exprofesional en enfermedades transmisibles de la oficina de OPS/OMS en Nicaragua), Pamela Mbabazi (asesora en monitoreo y evaluación del departamento de enfermedades tropicales desatendidas de la OMS), Martha Reyes, Jazmina Umaña, Gustavo Murillo y Lenin Pérez (profesionales de los programas de inmunizaciones y enfermedades infecciosas desatendidas del Ministerio de salud de Nicaragua), a Pierce Trumbo (consultor externo de la OPS/OMS) y a todos los participantes de los talleres de entrenamiento desarrollados entre 2015 y agosto de 2017.

#### Declaración

Las opiniones expresadas en este manuscrito son responsabilidad del autor y no reflejan necesariamente los criterios ni la política de la *RPSP/PAJPH* y/o de la OPS
